# Key design elements and mechanisms for nature-based healing in China’s National Parks: insights from expert interviews

**DOI:** 10.3389/fpubh.2026.1780352

**Published:** 2026-02-11

**Authors:** Xiangting He, Tong Yin, Yangnuo He, Chenyu Guo

**Affiliations:** 1 School of Art and Design, Xihua University, Chengdu, China; 2 China Southwest Architectural Design and Research Institute Corp. Ltd, Chengdu, China

**Keywords:** mental health, mood, nature therapy, public welfare, restorative environmental factors, tourism

## Abstract

**Introduction:**

National parks are increasingly expected to promote public health; however, healing-oriented design guidance remains fragmented and often detached from ecological red lines and measurable outcomes. Therefore, this study aimed to (1) determine the design elements consistently recognized as the key to enhancing nature-based healing effects; and (2) identify the priorities and bundling strategies among these elements and how can they be translated into actionable planning, design, and management recommendations, particularly in the Chinese context.

**Methods:**

We conducted semi-structured interviews (30–60 min) with 13 interdisciplinary experts involved in the national parks and near-natural landscapes in China. The transcripts were analyzed in NVivo using the six-phase thematic analysis by Braun and Clarke with a standardized codebook.

**Results:**

The following six themes emerged: (1) nature-based healing; (2) multisensory stimuli in the natural environment; (3) route rhythms; (4) management and operational strategies; (5) equity and needs for diversity; and (6) evidence base and outcomes evaluation.

**Discussion:**

This study contributes to an implementation-ready framework that links conservation limits to healing-oriented design and operations, and a scalable two-tier evaluation system. This advances practice by making restorative planning measurable, iterative, and accountable, helping managers translate multisensory and spatial rhythm insights into decisions that protect ecosystems while improving visitor wellbeing.

## 1 Introduction

Nature has long been regarded as a foundational environmental determinant of human health, providing material resources and generating health benefits through psychophysiological regulation, stress reduction, and social connectedness (1, 2). Against the dual backdrop of rapid urbanization and epidemiological transition, the global burden of chronic diseases, mental disorders, and suboptimal health continues to increase, prompting public health and environmental governance to view nature-based health interventions as cost-effective and sustainable strategies (3). Evidence of a robust positive association between natural contact and mental health has been consolidated in recent years. Moreover, supporting practices have expanded beyond early modalities, such as spa therapy, air cures, and heliotherapy, to contemporary forms, including forest therapy, horticultural therapy, and natural prescriptions (4, 5).

As large-scale conservation units safeguard natural and near-natural ecosystems, national parks are increasingly expected to contribute to ecological process maintenance and the enhancement of public health and wellbeing (6). Compared with typical urban green spaces, national parks often exhibit higher ecological integrity, greater landscape continuity, and more immersive natural experiences. Additionally, a growing body of research has demonstrated positive associations between national park visits and improved mental health, effective stress recovery, and enhanced subjective wellbeing (7–9). Nevertheless, current practice remains largely oriented toward landscape aesthetics or ecological safety and lacks an operationalized framework that translates design elements into measurable healing effects, thereby limiting the potential to maximize psychological restoration. In China, the national park system is in a phase of rapid expansion characterized by heterogeneous landforms, pronounced seasonality, ecological fragility, and high visitation, which places increased demands on the integration of healing-oriented design and management (10). Accordingly, identifying and systematizing key design elements and their underlying mechanisms at the national park scale is theoretically and practically warranted.

Since the proposition by Olmsted in the nineteenth century that green space can alleviate attentional fatigue and reduce everyday stress, ideas linking nature, attention, and health have become increasingly systematized. In 1989, Kaplan posited the Attention Restoration Theory (ART), arguing that environments characterized by soft fascination, being away, extent, and compatibility could restore directed attention and executive functioning (11). Complementing ART, the Stress Recovery Theory (SRT) posits that natural cues can reduce physiological arousal and stress through rapid affective and psychophysiological pathways (12). Building on these mechanisms, a growing body of evidence has shown that natural exposure is associated with improved objective indicators, including increased heart rate variability, decreased blood pressure, reduced skin conductance, and salivary cortisol, as well as more consistent subjective benefits, such as enhanced positive affect, reduced fatigue and anxiety, and improved performance on cognitive tasks (2, 9, 13). Neuroimaging and mobile sensing studies further suggest that natural stimuli are linked to reduced prefrontal load and optimized activity in emotion regulation networks, indicating dual timescales in restoration—rapid emotional recovery alongside slower cognitive recovery (14).

However, research examining the multilevel pathways through which natural contact exerts its effects is expanding and is actively debated. In forest therapy contexts, meta-analyses have indicated that even brief forest walks or stationary stays can reduce perceived stress and anxiety scores (15). Such benefits may emerge immediately after exposure and/or strengthen over time and tend to be greater in settings with low noise, moderate physical demands, and rich sensory cues compared to their counterparts (16). Meanwhile, “natural dose” research has primarily focused on parameters such as exposure duration, contact frequency, and spatial quality to develop analytical models to explain how doses shape biological systems and human health outcomes. Exposure that is too brief may be insufficient to trigger meaningful homeostatic changes, whereas excessively long durations or high loads may offset potential gains (17). Moreover, the therapeutic effects are substantially moderated by individual differences. Children, older adults, and people with suboptimal health often show greater sensitivity to natural exposure in terms of executive functioning, emotional stability, and sleep, compared to their counterparts. This indicates that age, physical capacity, psychological baseline, nature preference, and social use patterns may alter the magnitude and persistence of the effects (18, 19). Collectively, these findings suggest that nature-based healing does not arise from “nature” in the abstract, but depends on the fit between specific configurations of environmental elements and the characteristics of particular user groups.

Compared with the macro-level notion of “nature exposure,” specific design elements directly determine the intensity of stimuli that visitors actually receive and the quality of their experiences. Previous studies can be broadly synthesized as the joint effects of three sets of variables: spatial configuration, multisensory cues, and operational management. Trail gradient, curvature, and inter-node spacing shape the rhythm of physical and attentional loads. For instance, when walking–pausing–viewing sequences are deliberately orchestrated, outcomes such as perceived restoration and heart rate variability are more likely to improve (20, 21). Practice-oriented exploration in protected areas further suggests that a hierarchical network of main routes, secondary spurs, and quiet branches combined with viewpoints, contemplative stops, and waterside nodes can provide a graded supply of load and experiential intensity from low to high without breaching ecological and safety boundaries (22). For example, common landscape layout approaches in North America and Europe employ far–mid–near view-depth gradients to relieve crowding at peak nodes and reduce the perceived risk (23). In addition, accessibility standards and micro-rest spaces can expand the reach for older adults, families with young children, and visitors with lower physical capacity, thereby improving overall restorative benefits (24).

Multisensory design and operational management can amplify or attenuate spatial effects. For instance, soundscapes often explain emotional recovery better than single visual scene indicators (25); accordingly, quiet zoning, traffic noise shielding, and visitor capacity management have become priority management strategies. In popular canyons or waterfall areas, timed-entry reservations and one-way slow routes can simultaneously reduce noise and crowd interference, with empirical evidence indicating that lower perceived crowding frequently co-occurs with higher restoration (26, 27). Additionally, within wayfinding systems, shifting from information-dense formats to readable designs with moderate cognitive load, clear directionality, and emotion-friendly messaging can reduce disorientation and time pressure, and improve person–environment fit (28). Moreover, the combined embedding of waterside patches, fragrant planting bands, and tactile media at point–line–surface scales is often associated with increased heart rate variability, a more positive affect, and greater aesthetic pleasure (29). However, evidence on optimal “mixes” and maintenance costs remains limited. This is particularly salient for the national parks in China, where pronounced multidimensional landscape heterogeneity raises the unresolved challenge of dynamically balancing restorative benefits against ecological disturbances and safety risks.

Despite these advances, existing research has notable limitations in terms of external validity and implementation. First, many samples were drawn from urban green spaces or small experimental sites, constraining generalization to national park contexts characterized by long routes, strong seasonality, complex landforms and high visitation. Second, trade-offs have been insufficiently examined; for instance, enhancing human–wildlife interactions may enrich experience but also increase the risk of ecological disturbance. Third, mechanism measurement often emphasizes endpoint outcomes while lacking parallel observation of mediators such as perceived crowding, cultural meanings, and risk perception, leaving the causal chain from design stimuli to healing outcomes incomplete. Accordingly, systematic identification of design elements at the national park scale under conditions that integrate design, operations, and management and a clarification of their boundary conditions and implementation requirements is urgently required. Therefore, this study aimed to determine the following: (1) the design elements consistently recognized as the key to enhancing nature-based healing effects; and (2) identify the priorities and bundling strategies among these elements and how can they be translated into actionable planning, design, and management recommendations in the Chinese context.

This study interviewed domain experts to elicit practice-based judgments on healing-oriented design elements, addressing a key gap in the existing literature, namely, the weak linkage between aesthetic and ecological objectives and a coherent evidence chain for therapeutic outcomes, thereby underscoring the urgency of identifying critical design elements and clarifying their mechanisms. This study is expected to generate a context-sensitive prioritization and bundling strategy for design elements, providing implementable decision support for the multi-objective planning and management of national parks. Internationally, the findings of this study can broaden the empirical landscape of research beyond Western settings by testing and extending the boundary conditions of prevailing nature-based healing theories and articulating design principles with cross-regional transferability to inform the transition of national parks from primarily ecological protection to integrated public wellbeing functions.

## 2 Materials and methods

This study adopted a qualitative research approach and employed a design that combined semi-structured interviews with thematic analysis. Interviews were conducted using a pre-specified guide organized around core topics and open-ended questions to ensure that participants provided relevant information across key dimensions and enhanced cross-case comparability (30). Simultaneously, the semi-structured format allowed the interviewer to adjust the pace and probing depth in response to participants’ real-time feedback, using follow-up questions to move beyond the initial guide and capture rich, nuanced accounts of participants’ internal understanding and practical experiences (31). Thematic analysis is a well-established method for systematically identifying, analyzing, and reporting recurring meaningful patterns in textual data. In this study, we followed the six-phase framework—data familiarization, initial code generation, searching for candidate themes, reviewing and refining themes, defining and naming themes, and producing a report—by Braun and Clarke (32) which has been widely adopted because of its transparent logic and broad applicability. This stepwise procedure ensures that the findings address the research questions while maintaining analytical rigor, transparency, and traceability.

**Figure 1:**
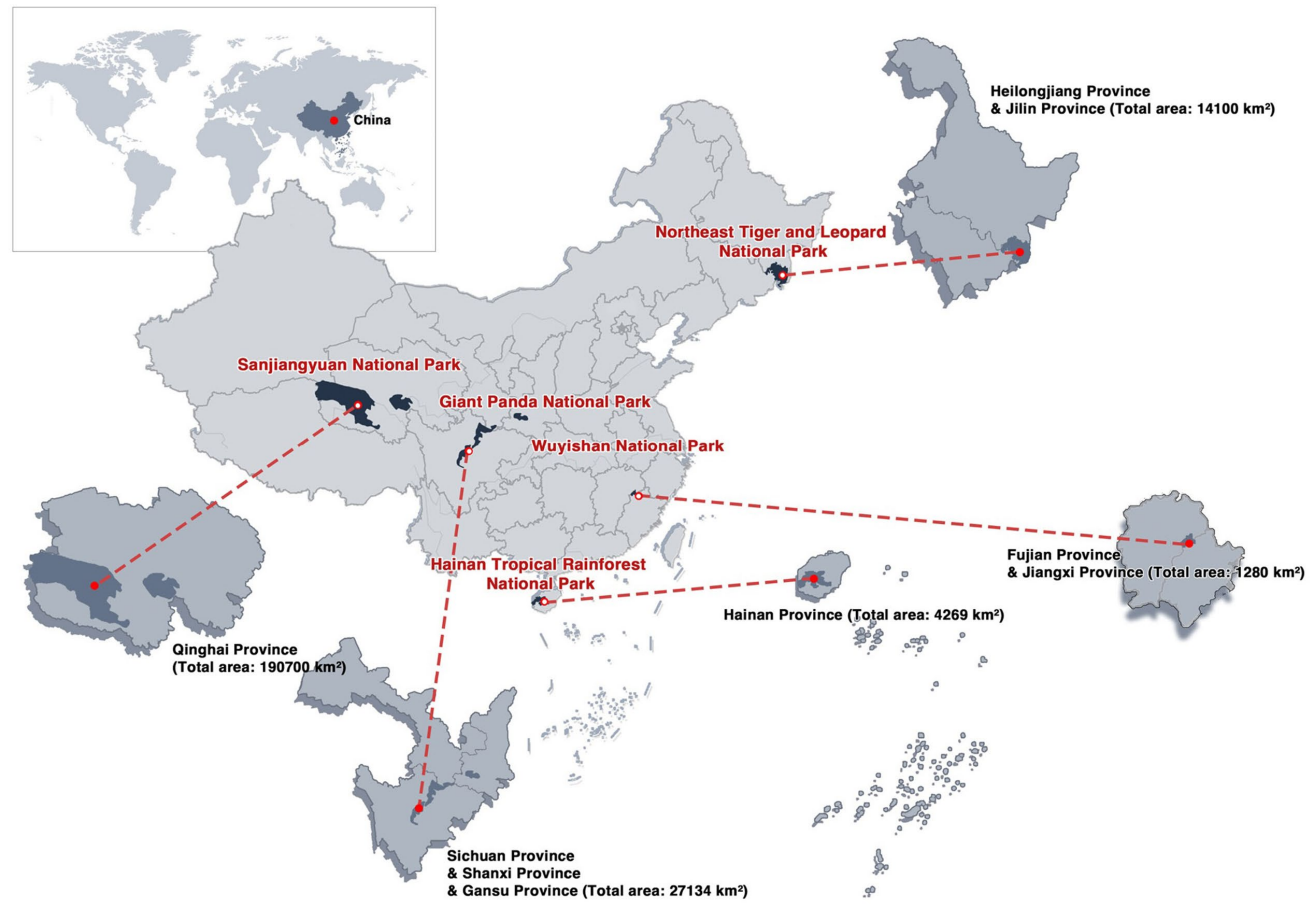
The distribution of China’s first batch of national parks. Source: drawn by the authors.

In China, the national park system has been designed to conserve ecosystems and natural heritage, and exhibits a large-scale, multi-type, and highly heterogeneous landscape configuration. The system spans diverse landforms, including alpine plateaus, temperate mountains, subtropical hills, and tropical islands, encompassing the alpine meadows and glaciers of Sanjiangyuan National Park, the mixed conifer–broadleaf forests and mountain gorges of Northeast Tiger and Leopard National Park, the subtropical montane evergreen broadleaf forests of Giant Panda National Park, the rainforests and stream valleys of Hainan Tropical Rainforest National Park, and the Danxia landforms interwoven with mid-subtropical forests in Wuyishan National Park (Figure 1). Most areas feature high vegetation cover and strong ecological integrity and are dominated by extensive contiguous natural forests, grasslands, and wetlands with a low proportion of built-up land and hardscapes. The disturbance intensity typically decreases from core conservation zones to recreational and service areas, preserving relatively authentic and immersive natural experiences. This stable spatial and managerial structure, which balances large-scale landscape patterns, intact ecological processes, and moderate recreation, offers a representative setting for examining how landscape elements are related to healing outcomes.

### 2.1 Participants

This study was approved by our institutional ethics review committee and written informed consent was obtained from all participants. The interviews were conducted in March 2025. Recruitment followed a staged strategy combining maximum-variation purposive sampling with snowball referrals. Specifically, we first mapped key expert strata across the planning–design–operation–health chain and recruited participants purposively to ensure heterogeneity in professional roles and perspectives. Snowball referrals were used solely to identify additional eligible experts to fill uncovered strata, rather than to expand the sample indefinitely. Recruitment and analysis proceeded iteratively, and recruitment was terminated when information saturation was reached—specifically when two to three consecutive interviews generated no new initial codes and no additional first-order themes or core design elements; an additional interview was conducted to confirm stability (33). In this study, first-order themes refer to the sub-themes (see Appendix 1 for the coding hierarchy), whereas core elements denote discrete and actionable healing-oriented planning/design/management features proposed by the experts.

Participants were recruited while maintaining diversity across age, educational background, occupation, and sex to enhance data heterogeneity and the transferability of findings. The inclusion criteria were: (1) direct involvement in any stage of national park planning, design, or evaluation; (2) publication of peer-reviewed work related to nature-based healing, forest therapy, or closely allied topics; and (3) at least 3 years of employment in a relevant management agency with responsibilities for health promotion, ecotourism, and recreation. In total, 13 experts in national parks and near-natural landscapes were included, representing interdisciplinary perspectives spanning planning and design, landscape ecology, operations and management, and mental health research (Table 1).

**Table 1:** List of interviewees.

No.	Age	Sex	Education	Occupation	No.	Age	Sex	Education	Occupation
1	31	Female	Doctor	Lecturer	8	28	Female	Master	Spatial designer
2	29	Female	Master	Rural planner	9	29	Male	Doctor	Researcher
3	55	Male	Doctor	Physician	10	59	Male	Doctor	Park manager
4	31	Male	Doctor	Lecturer	11	48	Female	Doctor	Physician
5	43	Female	Bachelor	Tourism manager	12	31	Female	Master	Landscape manager
6	52	Male	Doctor	Researcher	13	30	Female	Doctor	Researcher
7	29	Female	Master	Planner					

### 2.2 Data collection

**Figure 2:**
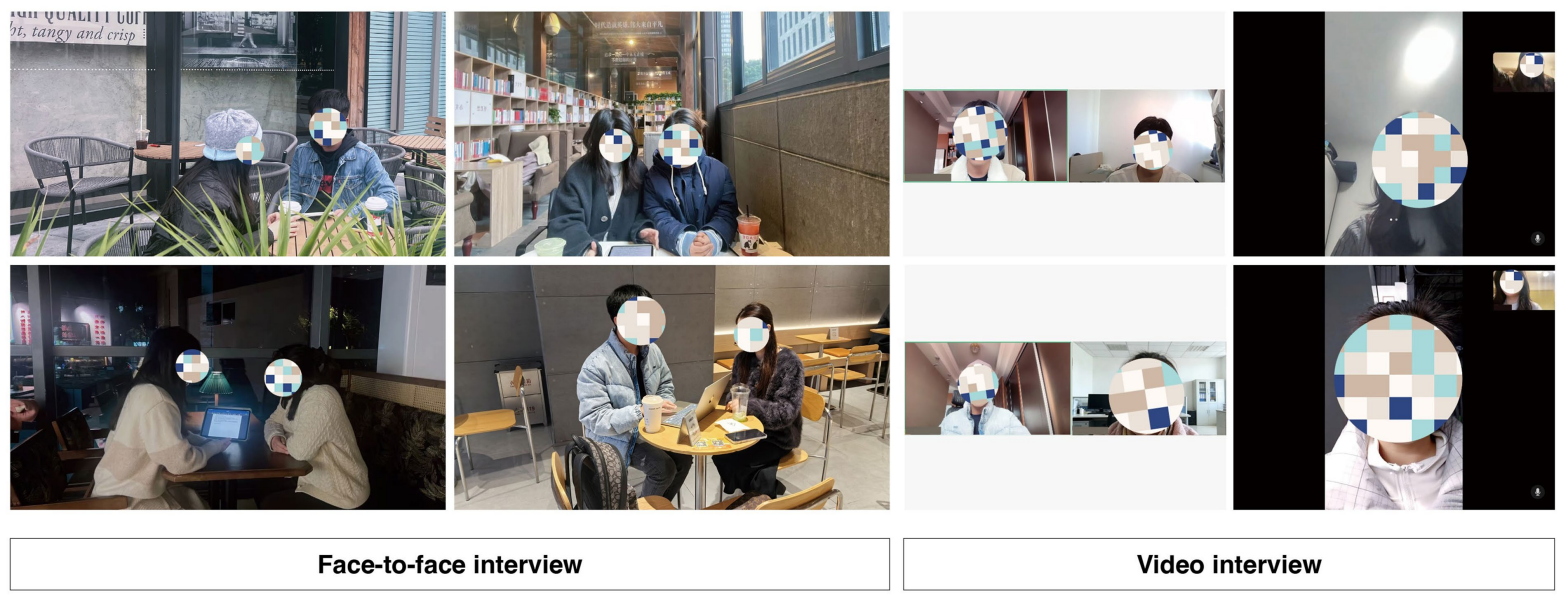
Interview scene recording. All respondents have consented to the portrait collection, and the author team has implemented privacy protection measures for the involved portrait content. Source: taken by the authors.

Each interview lasted 30–60 min and was conducted either via video conferencing or in person. Before the interview, the participants were informed of the purpose of the study and how the data would be used and handled. The interviews were audio-recorded in full, and the researcher concurrently produced field memos. Four participants were reluctant to be recorded; therefore, their verbal accounts were transcribed in real time using an iFLYTEK Office Notebook X3 Pro to address privacy concerns (Figure 2).

The interview guide focused on three categories of design elements: spatial and route configuration, multisensory cues, and operational management, and included modules for mechanisms and evaluation. These modules prompted experts to propose actionable indicators along a design stimulus, emotion, physiology, behavior, and health pathway, and to explain how user characteristics shape element priorities and trade-offs. The guide was pilot tested by two domain experts, and following the refinement of wording clarity, terminological consistency, and question sequencing, a finalized version was adopted to ensure cross-case comparability (Table 2). Additionally, the pilot step served to verify the adequacy of the planned expert strata and ensure that subsequent recruitment targeted complementary perspectives rather than redundant profiles.

**Table 2:** Interview outline.

Topic	Question
Interviewee background	Please briefly introduce your role and experiences in national parks/natural landscapes close to nature.
	Please share your significant discoveries during this process.
Space and path	In terms of spatial organization, which paths and node designs can most effectively, and consistency enhance the recovery experience?
Multisensory cues	Based on your experience, if we take the five senses as the starting point, which sensory experience do you think is the best in terms of importance and effectiveness (visual, auditory, olfactory, tactile, gustatory)?
	How can microclimate factors (wind direction, shade, sunlight exposure, perceived temperature) be integrated into the design of paths and nodes?
	How do you view the role and boundaries of the olfactory and tactile elements (fragrance corridors, barefoot paths, touchable media) in the healing process?
Operation and management	Which operational strategies can significantly enhance the recovery experience?
Mechanism chain and indicators	If you want to monitor the therapeutic effect in the project, what is the most feasible combination of indicators?
Context dependence and balance	For different groups of people (elderly, children, sub-healthy individuals, families/groups), which design or operational details need to be specially optimized?
	Please share a scenario of trade-offs: For instance, water accessibility improves the experience but raises risks of safety and ecological disturbance. How do you make a decision?
Implementation path and replicability	If healing-oriented is implemented as a project process, how should the key steps and responsible parties be divided?
Comprehensive judgment and ranking	Please rank the following elements according to their importance for the healing effect and briefly explain the reasons. Candidate list (can be added or deleted): path slope/curvature, node spacing/holding rhythm, field of view gradient, silence/low-speed zoning, capacity management, microclimate (shading/ventilation), naturalness of soundscapes, accessibility to water, olfactory/tactile media, continuity of accessibility for the disabled, readability of guidance, risk communication.
Policy and collaboration	Which provisions in the current norms or policies facilitate or hinder healing-oriented design and operation?
Ending	If you want to verify your current judgment within the next 3 years, what is the pilot project that you would most like to carry out? How would you evaluate the results?
	Are there any other questions that I have not addressed, which are crucial for the design and management of national park and natural healing?

### 2.3 Data analysis and procedure

The audio recordings were processed by two researchers using a combination of independent transcription and crosschecking by a research assistant. Transcripts were proofread verbatim, preserving participants’ tone and natural pauses to capture implicit meanings and intensity of expression. Coding was conducted using NVivo by two coders with interdisciplinary training in landscape planning and health. Before formal coding, two interview transcripts were used to complete two rounds of independent parallel pilot coding. Discrepancies were compared and resolved through discussion, resulting in a standardized codebook that specified code definitions, inclusion/exclusion criteria, and exemplar quotations. We adopted a hybrid deductive–inductive strategy, and the thematic analysis strictly followed the six-step procedure by Braun and Clarke.

Credibility and confirmability were strengthened through multiple strategies. First, triangulation was supported by a heterogeneous sample with diverse professional backgrounds and positions in the work chain, enabling cross-validation of perspectives. Additionally, where feasible and without collecting personally sensitive information, we also used non-interview materials (e.g., publicly available project documents and planning materials) as supplementary evidence. Member checking was conducted by returning theme summaries and key formulations to participants for confirmation and revision. We documented which suggestions were incorporated and the rationale for any non-adoption. In addition, an independent researcher who was not involved in coding conducted peer debriefing, interrogating theme boundaries and the plausibility of interpretations. We retained the codebook, theme development maps, and coding change logs to establish a comprehensive audit trail.

**Figure 3:**
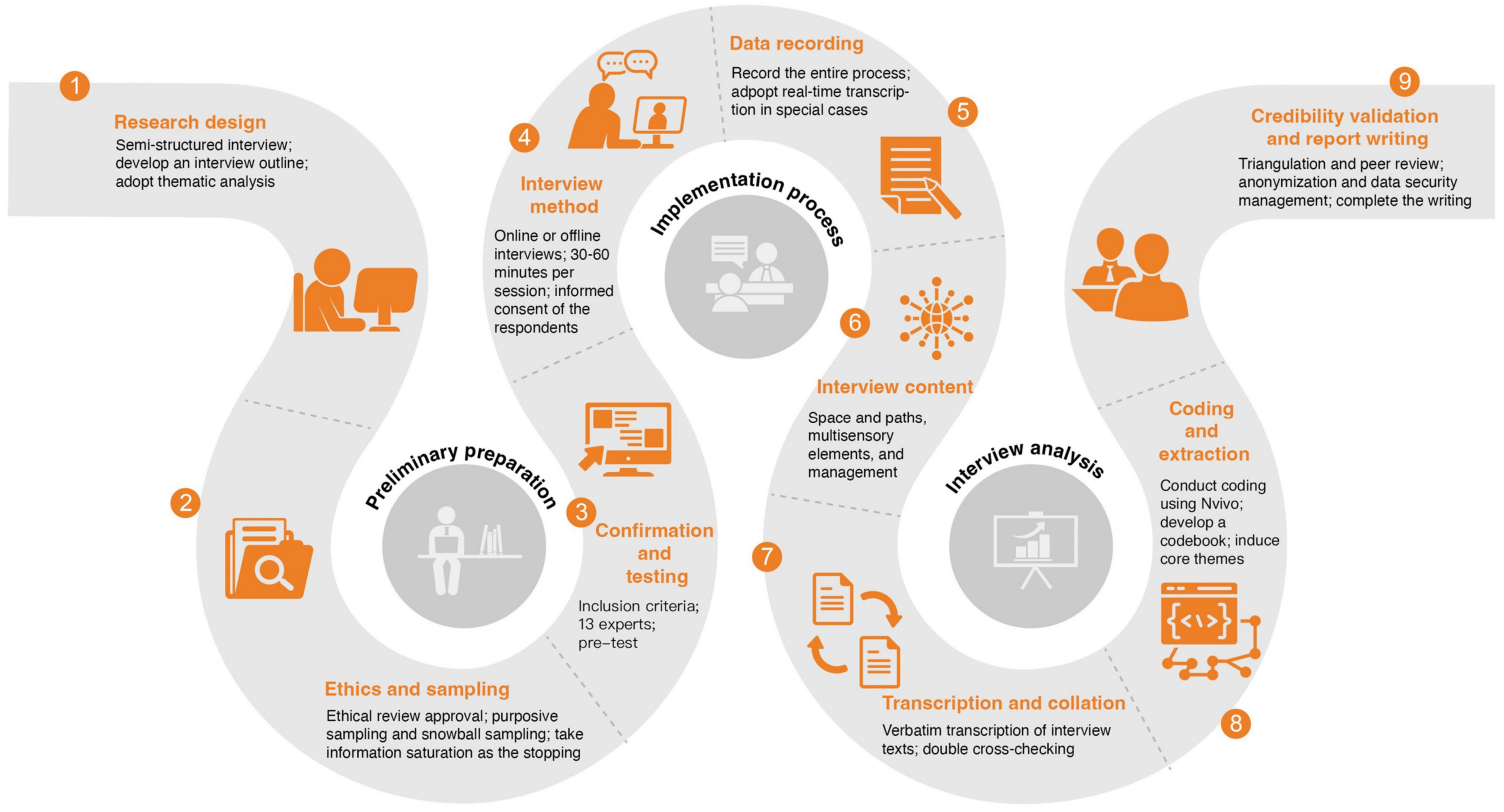
Research framework. Source: drawn by the authors.

Data management complied with the research ethics and confidentiality requirements. All audio files, transcripts, and analytical materials were de-identified and stored on an encrypted hard drive and an access-restricted cloud platform. The files were labeled using anonymous identifiers (N1–N13), and access was limited to authorized project members. All participant statements quoted in the manuscript were screened to remove information that could identify individuals or institutions, thereby minimizing privacy risks (Figure 3).

## 3 Results

Based on thematic analysis, the following six overarching themes were identified: (1) nature-based healing; (2) multisensory stimuli in the natural environment; (3) route rhythms; (4) management and operational strategies; (5) equity and needs for diversity; and (6) evidence base and outcomes evaluation. Collectively, these themes provide a structured overview of healing-oriented practice in China’s national parks as articulated by the experts. Specifically, Theme 1 captures the boundary conditions and value positioning under ecological conservation; Theme 2–3 describe the primary experience-enabling levels at the environmental and spatial/route levels; Theme 4 summarizes the operational and managerial conditions considered necessary to maintain these experiences in practice; Theme 5 highlights cross-cutting considerations of user heterogeneity and equity that shape how strategies and tailored; and Theme 6 concerns the evaluation-oriented evidence infrastructure that experts viewed as essential for monitoring and iterative improvement. The themes are reported in this sequence to reflect how participants commonly organized the issue from constraints to implementation and monitoring (Appendix 1).

### 3.1 Theme 1: Nature-based healing

The coding process for Theme 1 is as follows (Table 3):

**Table 3:** Coding framework for Theme 1.

Initial coding	Sub-themes	Theme	Definition
Natural healing is considered an auxiliary value	Natural healing as an auxiliary value	Nature-based healing	Within the management framework of national parks—where ecological conservation is the primary premise—natural healing is positioned as an auxiliary or secondary value. Its development should proceed in a moderate manner and must not cross ecological red lines, to balance human experience with environmental protection.
Natural healing has not become a core objective			
Healing is not explicitly mentioned in planning practices			
The general public does not recognize the healing value of national parks	Public awareness and concept popularization		
Interpreting healing from the perspective of ecological wellbeing for the people			
Upholding the ecological protection red line and the bottom line of inclusiveness	The boundaries and principles of healing practices		
The healing industry should be beneficial and harmless to local communities			

Most experts (12/13) adopted a cautious stance toward the role of nature-based healing in national parks. They noted that at the level of master planning, the concept of psychological healing was rarely articulated explicitly; rather, it was more often embedded within other objectives such as wellness tourism or improvements in public services. One planner (N2) stated that “nature-based healing is more of an add-on in relation to national parks; the core of national parks remains biodiversity conservation.” This observation suggests that within the current policy discourse in China, nature-based healing is typically subordinated to agendas such as ecological protection and community development rather than being positioned as an independent, leading objective in planning.

National park managers articulated clear boundary principles for nature-based healing. As N10 stated, “we cannot use so-called healing as a pretext for cross conservation red lines; conservation is not about isolating people from nature, but about organizing human–nature relationships in a more scientific and sustainable way.” The planner further delineated three managerial principles. First, any healing-oriented use must comply with zoning regulations and activities should not be promoted in areas unsuitable for opening, such as core conservation zones. Second, access should be reachable but controllable, providing opportunities for natural contact without increasing ecological pressure or safety risks. Third, managers should emphasize small-scale controls with large impacts, using carefully designed trails and nodes to redistribute flows and reduce disturbances rather than constructing large facilities. Several experts (e.g., N7 and N13) also highlighted the role of policy instruments in operationalizing healing objectives, such as entry-list systems that specify which industries and project types are permitted within national parks (4/13). Overall, national parks were recognized as having potential public wellbeing functions, and the interview data delineated the positioning and constraints of nature-based healing within the national park context.

### 3.2 Theme 2: Multisensory stimuli in the natural environment

The coding process for Theme 2 is as follows (Table 4):

**Table 4:** Coding framework for Theme 2.

Initial coding	Sub-themes	Theme	Definition
Environmental elements, including water bodies and floral resources enhance healing benefits	Positive healing landscape elements	Multisensory stimuli in the natural environment	It is emphasized that natural healing experiences can be strengthened through multi-sensory environmental elements (e.g., visual, auditory, and olfactory stimuli). The mechanisms through which different sensory cues—including pleasing landscapes, natural sounds, and fresh scents—contribute to psychological and physiological recovery are also examined.
Vision-dominated and multi-sensory integration	Vision-dominated and multi-sensory synergy		
Vision, smell, and hearing are important in that order			
The olfactory soothing effect of fresh air	The importance of soundscapes and olfactory experiences		
Microclimate comfort is second only to vision			
The balance between quiet natural sounds and noise			
Soundscapes have begun to be valued in planning			
The encounter with wild animals brings surprise and healing effects	The healing experience brought by wild animals		

Multisensory stimuli in the natural environment play an important role in emotional improvement and stress reduction (34). Visual and auditory factors were the most frequently emphasized stimuli, whereas olfactory and tactile cues were considered indispensable. Several participants (11/13) described the five-sense experience as the primary entry point for designing restorative settings. For instance, N1 noted that “existing studies often start from the five-sense experience; widely recognized positive landscape elements still center on water, flowers, and visual layering, as well as multisensory dimensions such as sound and smell,” and regarded these elements as a typical configuration associated with healing benefits.

The value of natural soundscapes was also highlighted repeatedly. The interviews suggested that many experts (9/13) viewed quietness and rhythmic natural acoustic environments as particularly influential in psychological recovery. N13 explained that “people often come to national parks for quiet… if your ears are still filled with human-made noise, it feels like you are still in the city.” In contrast, some participants (6/13) explicitly argued that sound could be more critical than vision because noise functions as a strong stressor, whereas gentle natural sounds lower vigilance and elicit relaxation responses. In this regard, an environmental–behavior researcher (N6) stated that “a continuous background of birdsong, water, and wind through the canopy pulls down people’s vigilance levels,” and ranked sensory importance as follows in order: hearing, touch, and vision. This view aligns with that of several medical and health-oriented experts (5/13). While visual cues provide immediate scenic appeal in national parks, where check-in tourism is common, overly strong visual stimulation may increase arousal and hinder relaxation (35). Conversely, sustained natural sounds and comfortable microclimatic conditions help to create a calming atmosphere and reduce autonomic stress, thereby providing a foundation for restorative effects.

Olfactory and tactile elements are generally considered to be beneficial supporting factors. Participants noted that floral scents and earthy forest smells can evoke pleasure and positive memories; although responses vary across individuals, a fresh woodland fragrance during appropriate seasons is often experienced as uplifting (e.g., N2, N5, and N7). Tactile cues, including bodily sensations of temperature, humidity, and wind, as well as touching trees or walking on soil and soft leaf litter, have also been described as integral to the restorative process (N1, N6). One practitioner shared an intervention involving walking with the eyes-closed to activate the non-visual senses and deepen natural engagement (N5), further supporting the value of multisensory participation in enhancing healing outcomes.

Finally, the experts emphasized that national parks have distinctive sensory advantages over urban parks. More original vegetation, diverse native species, and landscapes with fewer artificial traces in national parks compared to urban parks make it easier for visitors to engage physically and psychologically without common urban disturbances, such as noise, crowding, and visual clutter. Additionally, several participants (e.g., N5, N8) advocated minimizing human-made interventions, such as prioritizing natural soil trails over extensive hard paving and using native planting to create seasonal landscapes rather than introducing ornamental exotics to maintain authenticity and ecological function (5/13). N6 summarized that “healing-oriented design is often not about creating new spectacles, but about making existing restorative cues perceptible again, rather than being drowned out by noise and disturbance.” Collectively, these strategies preserve the wildness and immersion expected of national parks, strengthen human–nature connectedness, and enhance restorative experiences.

### 3.3 Theme 3: Route rhythms

The coding process for Theme 3 is as follows (Table 5):

**Table 5:** Coding framework for Theme 3.

Initial coding	Sub-themes	Theme	Definition
Spatial zonation and classification for differentiating the degree of openness	Zonal visitor flow control and order management	Route rhythms	By regulating the rhythm and frequency of spatial use in planning, sustained and balanced interactions between humans and nature can be promoted. This involves delineating functional zones and managing the volume and pacing of visitor flows, enabling people to meet experiential needs while safeguarding ecosystem recovery cycles.
Provide controlled accessibility			
Guide visitor flows through small-scale interventions			
Intensive control of key nodes plus flow dispersion at minor nodes	Stopping nodes and flow rhythm dispersion		
Allow intervals for ecological self-restoration	Ecological recovery cycles and visitor flow management		
Increased visitor flows bring environmental disturbances			
Identify healing elements based on stay duration	Pilot exploration and stopping behavior		

By scientifically designing route rhythms and stopover nodes, visitors with diverse abilities can comfortably, safely, and continuously engage with the nature. Appropriate control of trail gradients and curvature, frequent and well-sited resting points, and progressively unfolding vistas can further extend exposure time and reduce fatigue and tension, thereby supporting more stable and enduring restorative effects (36). Experts have repeatedly emphasized that restorative benefits depend on a continuous and steady experiential process that neither forces people to quit midway nor replaces relaxation with excessive arousal. Consequently, the trial design is widely regarded as a key determinant of healing outcomes. In this regard N6 explained that “when people enter nature, what they gain is a continuous set of experiences, rather than a single-point stimulus. Thus, restoration is more like lowering stressors, enhancing restorative cues, controlling disturbances, and organizing rhythm so that people are willing to stay.” This statement captures the core logic that an appropriate tempo enables visitors to get in and settle down, allowing stress to ease and recovery to gradually unfold.

Nearly all participants (12/13) highlighted the importance of the trail slope, continuity, and spacing of the remaining nodes. They noted that national parks often feature rugged topography. Therefore, without design interventions, many visitors may terminate their experiences because of fatigue or discomfort. Medical-background experts, such as N3 and N11, also underscored the concept of sustained exposure, arguing that restorative gains are closely linked to the time spent in nature (37, 38). Accordingly, routes should enable most visitors to walk comfortably for at least 20–30 min; otherwise, they may withdraw before reaching a relaxed state. Consequently, N3 proposed three design priorities to achieve this. First, failure rates can be reduced by avoiding overly steep or slippery surfaces and providing clear junction choices and exit options to prevent disorientation or trapping between progress and retreat. Second, a controllable stopover rhythm should be created. For instance, rest points should not be merely decorative but should genuinely invite pausing, with shade/rain shelters, seating, and modest scenic attractors that stop feeling relaxed rather than awkward. Third, perceived safety and predictability should be ensured through clear path boundaries, timely risk cues, and visible emergency facilities, so that visitors remain confident rather than tense about falling, getting lost, or encountering unknown conditions.

In addition to their physical feasibility, route rhythms also have psychological effects. Planning experts such as N9 noted that overly straight and monotonous routes or routes with excessive elevation changes can either exhaust interest too quickly or induce premature physical and mental fatigue. In contrast, gentle winding paths can slow the walking speed and sustain exploratory motivation. Alternating narrow and secluded segments with sudden openings at viewpoints can generate small expectations and micro surprises in various visual fields. After moving slowly along a shaded forest track, arriving at an open platform where distant mountains, lakes, or a sea of clouds comes into view can produce a strong sense of pleasure and accomplishment (N4). N9 argued that such rewards could offset hiking fatigue, restore vitality and encourage continued engagement.

Experts have also stressed on the need for microclimate regulation and a sense of refuge as prerequisites for sustained natural contact. Comfortable microclimate conditions help visitors stay long enough for restoration to occur. In contrast, persistent sun exposure, stuffy heat, or biting wind can undermine calmness, regardless of the scenic quality. Accordingly, participants recommended using vegetation and terrain to create localized cool conditions in summer, warm conditions in winter (e.g., sufficient shade and ventilation in summer), and windbreaks or sun-facing alignments in winter. N9 suggested increasing canopy cover and sheltered structures, leveraging evapotranspiration from tall trees for cooling, and using barriers to reduce wind. Similarly, N10 observed that many people cannot keep going or cannot stay because of heat, humidity, wind, rain, or unsafe surfaces and not because of the landscape. Therefore, details such as shade shelters, rain canopies, drainage, and anti-slip treatments are necessary to prevent climate-induced stress responses.

Meanwhile, complete openness may provide expansive views but little psychological shelter, leaving some visitors uneasy in overly exposed settings; conversely, dense forests with high enclosures can feel oppressive and heightened tension due to limited visibility (N8). Thus, practical solutions include adding sparse trees or small pavilions around open viewpoints to provide a sense of backing, and locating quiet lakeside rest nodes in slightly concealed positions to reduce disturbances while maintaining accessibility via a discrete spur path, balancing reachability and tranquility (39–41). Drawing on their design experience, N8 argued that spaces intended for immersive restoration should avoid maximal accessibility, as excessive access can attract crowds and disrupt quietness. Preferably, such locations should be moderately off the main flow to preserve an uninterrupted restorative atmosphere. This perspective was also echoed by planners and managers, such as N12, who reported placing healing-oriented areas in quieter zones away from the main entrances and peak flows using zoning and access controls to protect environmental quality.

Well-designed spatial rhythms and environmental comfort typically translate into longer stays and deeper interactions within a setting. Accordingly, multiple experts (7/13) have suggested that behavioral indicators, such as dwell time, walking speed change, and route completion rates, can serve as practical signals of whether restoration is occurring. When people feel relaxed and pleased, they tend to linger, spend time viewing, daydream, or spontaneously engage in sketching or meditation. In contrast, when they feel irritated or uncomfortable, they accelerate and leave. Therefore, N9 advocated monitoring the average dwell time at nodes and the proportion of visitors completing the route as objective evidence of whether spatial design achieves restorative goals. Similar observations have already been made in practice. For instance, N5 noted that some parents in the family groups they guided became so comfortable in the afternoon forest setting that they lay down and fell asleep, indicating profound relaxation and even light sleep, which are considered excellent signs of recovery. Others who typically experienced anxiety or insomnia reported markedly improved sleep at night following a full day in a natural setting.

### 3.4 Theme 4: Management and operation strategies

The coding process for Theme 4 is as follows (Table 6):

**Table 6:** Coding framework for Theme 4.

Initial coding	Sub-themes	Theme	Definition
Control visitor capacity and mitigate noise via greening to create a multi-sensory environment	Capacity management and visitor flow diversion	Management and operation strategies	Natural healing goals should be ensured through management and operational strategies, including visitor capacity control, zonal guidance, visitor flow organization, interpretive education, and staff training, to provide a high-quality healing experience while safeguarding ecological integrity, safety, and order.
Visitor flow limitation and reservations, visitor flow diversion, and guidance-based interpretation	Capacity management and visitor flow diversion/interpretive guidance and normative education		
Train staff and disseminate ecological rules			
Train local rangers to act as guides and achieve a win–win outcome	Staff training and local participation		
Regulate entrance order and manage service experience	Entrance management and integrated coordination		

The experts consistently agreed that soft operational and managerial strategies are essential safeguards for restorative experiences. An uncontrolled number of visitors, and deterioration of order, can quickly degrade even well-designed environments. N3 succinctly stated that “without capacity management, talking about healing is a luxury.” Visitor-carrying capacity has repeatedly been identified as the foremost determinant of the restorative quality of national parks. Crowding generates noise and physical degradation, and directly elevates psychological stress. Moreover, when surrounded by dense flows, people find it harder to feel relaxed and safe. Participants with planning practice (N2 and N8) observed that in some popular destinations in China, flow-control measures are largely nominal; even with reservation systems, the quota is often set too high, leaving sites overcrowded during peak seasons. Accordingly, several experts have called for scientific carrying capacity assessment models and strict reservation-based limits, even at the cost of reduced ticket revenues, to protect environmental quality and experiential comfort. N10 further framed capacity management as a policy-level foundation for institutional innovation and public benefit: “crowding brings noise, conflicts, and boundary violations at the same time; experience deteriorates and ecological pressure increases—flows must be kept within a reasonable level through reservations, time-slotting, zoning, and hotspot guidance.” This logic parallels public health approaches that manage population density to reduce accidents and transmission risks (42, 43).

Beyond the overall limits, visitor dispersion and circulation management were emphasized. N10 recommended separating fast-paced check-in routes from slow and quiet restorative routes. For example, a direct, efficient line can serve visitors who prioritize taking photos at major attractions, whereas those seeking calm walking can be guided onto secluded trails to reduce mutual interference. N3 noted that “many people are not unwilling to slow down—they are carried along by the flow.” Therefore, providing optional slow/quiet lines enables individuals with restorative needs to decelerate and sustain their engagement. Additionally, from a hotspot perspective, time-limited stopping and rotational viewing can prevent visitors from monopolizing premium vistas and causing bottlenecks (N10). In this regard, N6 described a complementary strategy that combined strong control at iconic nodes (centralized management and strict limits) with multiple low-stimulus stopovers along the route to distribute rhythm and disperse crowds. Collectively, these approaches highlight the value of fine-grained circulation organization in maintaining restorative order.

Interpretation style and content were identified as another critical operational lever. Conventional scenic site interpretation often aims to deliver information and direct must-see check-ins. This interpretation can be directive, information-dense, and task-oriented, keeping visitors in a performance mode rather than in a relaxed state. In contrast, healing-oriented interpretation prioritizes guided awareness and cognitive unloading, for instance, inviting visitors to close their eyes and listen quietly for one minute (N3) or offering brief embodied prompts, such as deep breathing or gentle stretching (N11). When health prompts are integrated with conservation reminders and safety information in concise and empathetic messages, they feel supportive rather than intrusive, which strengthens perceived care, trust, and safety. N11 stressed that “restoration can occur only when a sense of safety is established.” Additionally, N10 and N6 suggested reforming loudspeaker-based guidance (e.g., requiring headset interpretation for group tours) to avoid acoustic disturbances. They also advocated unified signage and rule communication at entrances and key locations, clearly stating behavioral restrictions alongside available services and emergency support. Furthermore, N10 noted that the goal is to create an environment that is orderly but not oppressive, where visitors can see the rules, understand the rules, and are willing to follow them, maintaining a sense of freedom with a reliable safety net, thereby enabling relaxation and immersion in nature.

Finally, the experts emphasized that developing and implementing operational strategies requires cross-departmental coordination and continuous data feedback. N10 noted a prevalent tendency toward building more and operating less and argued that standards and collaborative mechanisms for healing-oriented fine management remain underdeveloped. In this regard, standardization is required to clarify departmental responsibilities and protocols for capacity control, patrolling, and interpretive education. However, a real-time monitoring and dispatch system should be established that leverages intelligent sensing to track visitor volume, noise, and environmental parameters, promptly detect problems, and adjust measures accordingly. Meanwhile, N6 and N10 highlighted the importance of a “monitor–analyze–dispatch–feedback” closed loop. For example, if monitoring indicates rising noise levels on a trail alongside declining restoration scores, managers could tighten entry limits or increase dispersal. In case of repeated boundary violations and loud behavior in a quiet zone, enhanced patrolling or improved interpretive prompts may be required. Such evidence-based adaptive management allows operational strategies to be refined iteratively and helps translate restorative goals into consistent on-the-ground outcomes.

### 3.5 Theme 5: Equity and needs for diversity

The coding process for Theme 5 is as follows (Table 7):

**Table 7:** Coding framework for Theme 5.

Initial coding	Sub-themes	Theme	Definition
Differentiate strategies for distinct age groups and pay attention to the health background of the elderly	Design requirements for specific groups	Equity and needs for diversity	Design and service provision should attend to the natural healing needs of diverse groups (e.g., older adults, children, people with disabilities, and those from different cultural backgrounds). Under the premise of ecological protection, inclusiveness and equity should be strengthened to ensure that natural healing benefits all population groups.
Differentiated arrangements for the elderly, children and group tourists			
Trade-off between barrier-free accessibility and ecological protection	Balance between barrier-free access and ecological protection		
Provide inclusive healing services	Principles of inclusiveness and equity		
Cultural backgrounds shape the mindsets of healing experiences	Cultural contextual differences		

National park visitors are highly heterogeneous in terms of age, health status, and visit motivations. Therefore, their design and operations must accommodate the needs of different user groups to realize the broad potential of nature-based healing. Older adults have been repeatedly identified as a priority population, which often has a strong demand for nature-based wellness, with some retirees even relocating long-term to well-known health destinations. However, they are among the most vulnerable groups due to limitations in physical capacity, sensory function, and baseline health. N11 summarized that “older adults have a high risk of falls and a low fatigue threshold.” Thus, the minimum requirements for this population include continuously accessible trails, dense and appropriately spaced rest points, adequate shade and wind protection along routes, convenient toilets, and reliable emergency access. Several experts (e.g., N3, N6, and N12) emphasized the necessity of accessibility measures such as ramps in lieu of steps, handrails, non-slip surfaces, and wheelchair access to major viewpoints, without which older adults and people with disabilities are effectively excluded from restorative experiences. Additionally, N12 cautioned that some destinations marketed as wellness sites are not environmentally suitable for prolonged stays by older visitors, underscoring the need for medical posts and emergency response systems to mitigate acute health events. These provisions form a precondition for older visitors to feel secure and willing to engage with nature.

Children also comprise a special group. Although naturally curious and active, they often lack safety awareness and may deviate from designated routes or engage in risky behaviors. Therefore, experts recommend short, exploratory, yet controllable loops or activity zones. For example, a circular adventure trail near a campsite could allow parents to wait at the starting point while children complete scavenger hunting or observation tasks and return to the same point. This would remain within parental oversight and avoid confusing junctions, satisfying the exploratory tendencies of the children while reducing the risk of being lost. Drawing on practice, N5 noted that a key goal for family groups is to strengthen parent–child relationships and foster children’s interest in nature, which is often best activated through gamified engagement. Accordingly, healing-oriented programs can incorporate nature games and interactive tasks (e.g., locating specific plants or observing insects) or story-based immersive experiences that allow children to relax, learn, and play, while providing parents with an opportunity to share their experiences and disengage from mobile devices. Notably, children’s participation should not compromise other visitors’ needs. For instance, noisy group activities may disturb those seeking quietness. Therefore, experts suggested temporal or spatial separation—either designating dedicated zones for children’s activities or scheduling such programs during low-visitation periods—to balance competing demands.

Sub-healthy groups, such as individuals with chronic conditions or high psychological stress, have also been identified as important targets for restorative designs. Compared to general visitors, these groups often seek quieter and more private settings and may avoid large group activities that increase social pressure or physical burden. This implies a need for refuge spaces that support solitary relaxation, for example, a small, secluded forest platform that accommodates one or two people, with screening vegetation or structures that are reduced. Circulation design can also provide short, returnable loop options so that visitors with limited stamina can return at any time, rather than being forced to complete a full route (N11). This aligns with a core principle in public health interventions—enhancing autonomy and choice to reduce psychological burden and improve participation (44).

In addition to individual differences, the needs of social groups also diverge. Family visitors often travel for three generations, making it challenging to reconcile children’s energy levels, adults’ interests, and older adults’ comfort. N6 and N11 emphasized that compatibility design and conflict management are central to family contexts. One approach is to place a fenced nature play area adjacent to an older-adult tea/rest zone using plants to buffer noise so that older adults can rest while still supervising their children. Large numbers and synchronized behaviors can generate substantial acoustic and movement disturbances for organized groups (e.g., corporate outings and student trips). Experts recommended time-slot reservations and staggered scheduling to prevent large groups from entering quiet zones simultaneously with independent visitors or limiting group activities to designated areas, for example, allocating an open lawn for group games and photos while reserving forest trails for small groups or individuals seeking quiet walking (N10).

Equity considerations also require the attention of groups that may otherwise be overlooked, such as local community residents and low-income visitors. N10 argued that national parks must benefit people, including not only distant tourists but also surrounding communities. Healing-oriented programs could therefore offer dedicated time windows or discounted access for local residents, enabling them to share the health benefits of nearby nature and potentially strengthening community support for conservation. Other participants noted that at present, relatively few people travel specifically for nature-based healing, and many obtain relaxation incidentally through leisure travel. This points to the need for public communication and education to raise awareness of restorative benefits and lower participation barriers so that people from diverse backgrounds are willing to try. In practice, this can be achieved by providing a portfolio of options—from movement-based programs such as tai chi or yoga, knowledge-oriented nature health walks, and silent forest meditation trails—so that different visitors can find accessible pathways to engage with nature in ways that fit their preferences and capacities.

### 3.6 Theme 6: Evidence base and outcomes evaluation

The coding process for Theme 6 is as follows (Table 8):

**Table 8:** Coding framework for Theme 6.

Initial coding	Sub-themes	Theme	Definition
Transform qualitative research into quantitative research and verify the therapeutic effects of blue-green spaces	Evidence accumulation in natural healing research	Evidence base and outcomes evaluation	It is emphasized that natural healing practices should be grounded in scientific evidence and that outcomes should be monitored and evaluated. This includes developing quantitative indicators, employing experimental and observational methods, and establishing an integrated monitoring and evaluation system to verify and improve the effectiveness of natural healing.
Distinguish between two-tier outcomes: immediate recovery and long-term health benefits	Monitoring indicators for healing outcomes		
Small-scale experimental comparison vs. macro-level correlation analysis			
Comprehensive evaluation combining subjective questionnaires, behavioral data, and physiological monitoring			
Behavioral data are more objective and reliable, while physiological and subjective indicators are subject to limitations	Trade-off between behavioral and physiological indicators		
A two-tier evaluation system comprising universal governance indicators and experience-health indicators	Exploration of indicator system construction and standardization		
Conduct preliminary demonstration with macro-level big data and then guide in-depth micro-level research			
The healing effects of actual projects lack tracking and verification	Limitations of evaluation practices		
Healing effectiveness indicators have not yet been specified at the planning stage			

Discussions on how to demonstrate the effectiveness of nature-based healing and how to evaluate the performance of design elements are particularly intensive. Several researchers and physicians (6/13) have argued that nature-based healing must be grounded in scientific evidence rather than aspirational ideals to gain broad acceptance among policymakers and the public. As N3 challenged, “What do you want to change, and how will you prove that it has changed?” This is a reminder that both researchers and practitioners need clearly specified intervention targets and rigorous methods to verify whether these targets are achieved. This theme reflects an emerging trend in landscape architecture and public health toward moving from qualitative experience to quantifiable, evidence-based justification (45–47).

Many experts (7/13) have described the ongoing efforts to strengthen the evidence base. N1 noted that domestic studies are increasingly aligned with international approaches, shifting from early questionnaire-based qualitative studies to more quantifiable and verifiable systems that incorporate meta-analyses and wearable sensing. N8 reported integrating subjective psychological scales with objective physiological data, for example, measuring perceived meaning in life while simultaneously recording skin conductance and heart rate to corroborate psychophysiological improvement during natural experiences. N9 described VR experiments that manipulated vegetation density and collected blood pressure, heart rate, and eye-tracking data, showing that affective and physiological responses fluctuate with environmental changes. Such studies strengthen causal inferences about the environment–health links and provide actionable design guidance—for instance, suggesting that moderate vegetation density may better alleviate negative affect, whereas overly dense planting may induce oppression, and overly sparse planting may feel lifeless.

Multiple experts (10/13) have advocated a multi-level, multi-dimensional indicator system to monitor restorative outcomes. Accordingly, synthesizing their views, the system should include at least four domains:

First, subjective psychological indicators were assessed. Self-reports offer a direct assessment of restoration and can be captured through brief instruments that measure perceived restoration, positive affect, and stress. N6 and N11 recommended administering short questionnaires before and after park visits or around key nodes to detect within-person changes. Additional items such as perceived safety, tranquility, crowding, and satisfaction can help capture a broader experiential profile.

Second, objective physiological indicators are used. Heart rate and HRV are widely considered the most feasible primary measures given their sensitivity to stress/relaxation responses and the maturity and portability of current devices. Where feasible, blood pressure and electrodermal activity may also be measured. However, N6 cautioned against introducing overly complex equipment early to avoid participant resistance. N11 noted that confounding factors such as physical activity should be considered to prevent misattribution.

Third are behavioral and participation indicators. This domain is strongly emphasized because behavioral data illuminate how design affects outcomes. Common measures include location traces, dwell time, changes in walking speed, and route completion. Longer dwell time, slower pace, and higher completion rates along restorative routes may indicate an effective design, whereas frequent early exits or detours may signal problems. N11 proposed brief momentary assessments at key nodes and linked real-time mood/stress reports to location data to pinpoint segments that elicit positive or negative responses.

The fourth are the environmental and operational indicators. These inputs to the restorative mechanism include microenvironmental measures (e.g., noise level, temperature, humidity, wind speed, illuminance, and air quality) and operational records (e.g., visitor peaks, complaints, and rescue incidents). Such data help explain the temporal or spatial deterioration in restoration; for example, heat waves or sudden noise increases may depress restoration ratings. N6 and N10 stressed integrating environmental and management data into the monitoring system and analyzing them jointly with outcome measures. If restoration scores systematically decline as noise rises on a given route, managers are more likely to prioritize noise-reduction actions.

Overall, the experts conceptualized the indicator system as three linked data streams—environmental inputs, experiential processes, and psychophysiological outputs—captured via sensors, questionnaires, and wearables. N10 further recommended a two-tier architecture: Tier 1 comprises routine indicators that all parks can monitor (e.g., visitor volume, noise, dwell time, and other environment/behavior metrics), whereas Tier 2 involves a deeper collection of experience and health indicators (e.g., psychological scales and HR/HRV) in pilot parks to validate restorative effects under small-scale compliant protocols. This structure balances scalability with scientific rigor.

Crucially, several experts (9/13) emphasized the use of an evaluation system for iterative feedback and mechanism building rather than archival documentation. N6 and N10 advocated a closed-loop approach—monitoring, analysis, dispatch, and feedback—embedding restorative indicators within a unified park monitoring network that links directly to management decisions. For example, if one restorative trial consistently outperforms the other in terms of restoration ratings and dwell-time patterns, managers can compare spatial features, extract transferable principles, and apply them to other routes. Conversely, if a node performs poorly, design and operations can be adjusted promptly. As N6 noted, what should be replicated is not a fixed form but the logic of goals, parameters, operations, and evaluation.

Finally, the evidence base concerns the validation of the mechanisms. Some experts (e.g., N3 and N11) cautioned against vague mechanism claims and called for testable causal pathways (6/13). N11 explicitly suggested hypothesizing that spatial and operational conditions provide restorative cues and low-stress contexts, which influence affect, stress levels, and behaviors via mediators such as perceived safety and attentional restoration. and then used multi-source data to test whether these mediators are significant. N3 also highlighted the need to disentangle the effects attributable to natural exposure from those driven by physical activity to avoid conflated causal attribution. These reflections point to the need for more rigorous quasi-experimental or controlled designs in future studies, enabling the field to progress from anecdotal observations to statistically robust evidence.

## 4 Discussion

### 4.1 Differences in primary sensory preferences across professional backgrounds

Notably, practitioners directly involved in on-the-ground park construction, such as planners and designers, tended to prioritize vision as the primary sensory channel for restoration. A design research participant (N1) further argued that approximately 80% of human information acquisition relies on vision, making visual effects more immediate and salient, and thus assigning greater weight to visual stimuli under comparable conditions. By contrast, experts from the medical and management domains placed greater emphasis on the auditory dimension. These cross-professional differences reflect variations in professional training, role objectives, and perceptual strategies (29). For instance, planning and design professionals typically foreground visual landscape-making (an aesthetics-oriented approach), whereas physicians, park managers, and scholars emphasize sound and quietness (a health-effect-oriented approach). This divergence is not contradictory; rather, it underscores the need for multisensory integration in restorative design and management.

### 4.2 Relationships and mechanistic pathways between themes

**Figure 4:**
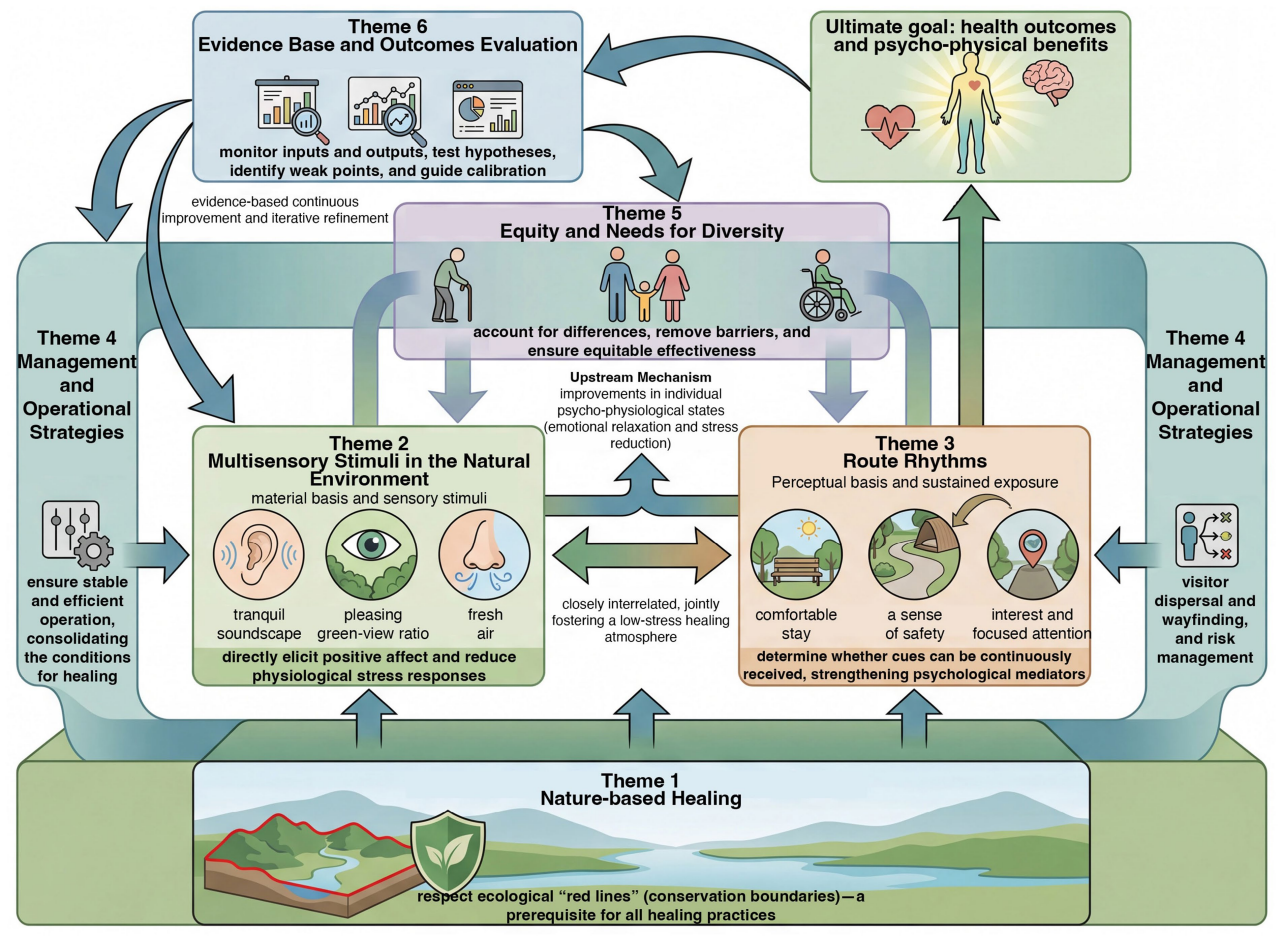
Healing mechanism. Source: drawn by the authors.

Building on the six themes reported in the Results, this section refines the descriptive findings into an integrated pathway model (Figure 4) by clarifying the functional roles of each theme and the relationship types that connect them. The synthesis highlights: (i) sequential enabling links from conservation constraints to experience-oriented design levers and then to health-related outcomes; (ii) reinforcing links whereby operations and management activate and stabilize on-site experiences under real visitation dynamics; (iii) a cross-cutting moderation effect of user heterogeneity and equity considerations on access, safety, and effect strength; and (iv) an evidence-driven feedback loop through which evaluation informs iterative adjustment. Within this structure, Theme 1 sets the non-negotiable ecological boundaries and governance principles; Themes 2–3 constitute the primary levers shaping restorative cues and the rhythm/dosage of nature engagement; Theme 4 specifies the operational conditions that sustain these levers in practice; Theme 5 qualifies the pathway across diverse populations; and Theme 6 closes the loop by translating outcomes into monitoring indicators and adaptive optimization. Overall, this synthesis translates the results into a coherent causal architecture, making cross-theme relationships explicit and actionable rather than merely parallel.

Specifically, Theme 1 provides the boundary conditions and value orientations for the entire mechanism. This ensures that any healing-oriented practice must respect the conservation red lines; only under this premise can other themes be meaningfully advanced. At the environmental and spatial levels, Themes 2 and 3 are closely coupled, jointly shaping the material and perceptual bases for restoration. Theme 2 emphasized the quality of sensory stimuli in natural settings, which is critical for eliciting positive affective responses. For example, quiet and aesthetically pleasing environments provide restorative cues such as gentle soundscapes, visually pleasing greenery, and fresh air, which can reduce physiological arousal and subjective tension. Theme 3 highlighted how spatial rhythms determine whether visitors can sustain sufficient exposure to these cues. As several experts (8/13) have summarized, environmental and spatial design together create low-stress conditions and an atmosphere rich in restorative cues. By enhancing psychological mediators such as perceived safety, comfort, and engaged attention, these conditions further translate into emotional relaxation and stress reduction. From a pathway perspective, Themes 2 and 3 primarily act on changes in individuals’ psychophysiological states, and thus constitute the upstream segment of the healing mechanism.

Theme 4 functions analogously to assurance and amplification for Themes 2 and 3. For instance, capacity control reduces disturbances such as crowding, anthropogenic noise, and visitor dispersion, route management optimizes visitor–environment interaction patterns, and interpretive guidance and risk management strengthen psychological safety and nature awareness. These measures directly consolidated the restorative conditions created by Themes 2 and 3. In this sense, Theme 4 operates at a more macro- and system-level by regulating external factors that shape human–environment interactions. One expert offered a particularly apt metaphor: “space is the carrier, operation is the trigger, management is the stabilizer, and evaluation is the calibrator.” Here, the stabilizer corresponds to Theme 4: Only when operations and management are well governed can restorative experiences remain stable and consistent, rather than fluctuating sharply with visitor volume or seasonal changes.

Theme 5 cuts across the preceding components and provides a basis for individualized adjustment within the mechanism. This underscores that, when applying strategies from Themes 2, 3, and 4, user heterogeneity must be addressed; otherwise, the healing mechanism may fail for certain groups or even produce adverse effects. For example, without additional accessibility and safety provisions, older adults may be unable to enter even the most beautiful settings or feel anxious and unsafe. For children, environments that are overly quiet or monotonous may lead to boredom and interrupt parents’ opportunities for relaxation. This indicates that user characteristics function as moderators within the pathway, shaping the magnitude and persistence of the effects for different populations. Through the lens of Theme 5, the earlier mechanism can be further specified: for each target group, the restorative cues–psychological mediators–outcomes chain must be re-examined to identify group-specific influences, and design and services should be adjusted to remove barriers and ensure the fairness and inclusivity of benefits.

Finally, Theme 6 provides the feedback loop and optimization driver for the entire mechanism. Without an evidence-based evaluation, the preceding aspirations remain hypothetical and lack a basis for sustained improvement. The multilevel indicator system emphasized in Theme 6 can test both the assumed mediators and causal links in the pathway and reveal weak points in real-world implementation. More importantly, evidence-based evaluation enables timely calibration for managers and designers, in which elements should be strengthened and adjusted measures can be guided by data. In other words, the feedback cycle driven by Theme 6 ensures that the healing mechanism adapts to dynamic conditions and continuously refines itself.

Taken together, the themes have an integrated mechanism chain for nature-based healing in national parks. Under conservation constraints, multisensory environments and spatial rhythms are optimized to improve visitors’ immediate psychophysiological states, operational and managerial measures maintain stable conditions and orderly experiences, diverse groups are enabled to participate appropriately, health-related benefits are realized, and evaluation data are fed back into iterative improvements in design and management. This process articulates clear causal logic. This mechanism aligns with established theories in environmental psychology. For example, Kaplan’s ART emphasizes that features such as soft fascination in natural environments facilitate attentional recovery, and the present findings specify how reducing noise and crafting a comfortable rhythm can operationalize such features (48). Ulrich’s SRT highlights that safe and pleasant natural scenes can elicit physiological relaxation responses, and the expert insights in this study place perceived safety and comfort at the center while proposing concrete strategies to reduce stressors (49). In this sense, the proposed pathway provides a practice-oriented elaboration of existing theory (50–52) and offers integrative validation of nature-based healing mechanisms within the context of China’s national parks.

### 4.3 Limitations and scope of future research

Despite the positive outcomes, this study has some limitations. First, the participants were primarily professionals; therefore, their perceptions and accounts may reflect a supply-side and elite perspective and may not fully capture ordinary visitors’ preferences or experiential heterogeneity. Accordingly, the findings more strongly represent planning and management logic with comparatively limited coverage of demand-side perceptions. Consequently, future research should incorporate systematic surveys of general visitors, *in situ* feedback, and behavioral data to assess the alignment between expert recommendations and visitors’ actual responses. Second, most experts were based in China. Although many of them have demonstrated an international outlook, the findings are inevitably embedded in a particular cultural context and governance system. Therefore, transferring these findings to other national park systems, where management arrangements and visitor norms may differ, may require contextual adaptation. Finally, given the interview-based qualitative design, some causal linkages among themes remain largely theoretical, despite efforts to strengthen interpretations through triangulation with evidence and prior literature. Hence, future studies should develop targeted empirical studies along key element–mediator–outcome pathways to test specific mechanisms and enhance the robustness and generalizability of the conclusions.

## 5 Conclusion

This study conducted a systematic and rigorous thematic analysis based on expert interviews, identifying key design elements for nature-based healing in national parks and clarifying the underlying mechanisms. The findings provide operationally actionable implications for subsequent planning and design practices as well as policy development. These implications distill expert experience and judgment into a clear action framework to achieve health gains amid the inherent tension between conservation and use.

The key findings are as follows. First, advancing nature-based healing in the context of national parks must adhere to the non-negotiable principles of ecological conservation priorities. The selection, configuration, and application of any design element should be premised on maintaining ecological integrity and system stability and avoiding short-term experiential enhancement at the expense of long-term ecological degradation. Secondly, multisensory integration is a central factor in eliciting restorative effects. High-quality visual scenery, rich yet low-disturbance natural soundscapes, clean air, and vegetation-related scents can serve as salient cues that trigger emotional repair and physiological relaxation, and should be systematically embedded in spatial organization and material strategies. Third, trails and nodes are core mediators that translate environmental cues into restorative experiences, and ultimately, restorative outcomes. Healing principles should be integrated into trail hierarchies, rhythm-based node layouts, and progressive landscape sequences, so that natural contact remains continuous, accessible, and perceptible across time and space. Fourth, management measures are as critical as design measures and should be integrated as a unified strategy within the healing mechanism, including capacity and crowding management, zoned and route-based operations, interpretive systems, and program planning to reduce ecological pressure and experiential disturbance while enhancing safety and accessibility. Fifth, differentiated priority orders and configuration logic should be established for the distinct user segments. Under resource constraints, national parks can prioritize elements that best match the health goals and usage patterns of the primary target groups, thereby improving cost-effectiveness and equity. Sixth, an evaluation framework that integrates research and management should be developed and institutionalized as a routine component of nature-based healing governance. Key elements and mechanisms should not be treated as static conclusions; rather, they evolve through continuous monitoring, feedback, and iterative optimization. Over time, cumulative evidence can reveal additional critical elements and enable more refined explanations and adjustments of the mechanisms identified in this study.

## Data Availability

The raw data supporting the conclusions of this article will be made available by the authors, without undue reservation.
