# Supplementary material for: Key design elements and mechanisms for nature-based healing in China’s National Parks: insights from expert interviews
Source: Front Public Health. 2026 Feb 11;14:1780352. doi: 10.3389/fpubh.2026.1780352 (PMC12932463; doi:10.3389/fpubh.2026.1780352)
Supplement: Supplementary file 1 [file Table_1.DOCX]

**Appendix 1. Results of Thematic Analysis**

| **Representative Statements** | **Initial Coding** | **Sub-themes** | **Themes** | **Definitions** |
| --- | --- | --- | --- | --- |
| N1: “The primary mission of national parks remains ecological conservation, while the healing function is more of an auxiliary value.” | Natural healing is considered an auxiliary value | Natural Healing as an Auxiliary Value | **Nature-based Healing** | Within the management framework of national parks—where ecological conservation is the primary premise—natural healing is positioned as an auxiliary or secondary value. Its development should proceed in a moderate manner and must not cross ecological red lines, to balance human experience with environmental protection. |
| N2: “Even so, the role of 'healing' will not be overemphasized. The connection between natural healing and national parks is more of a passing reference, and the core purpose of national parks remains focused on biological conservation.” | Natural healing has not become a core objective |  |  |  |
| N13: “Overall, 'natural healing' is not a particularly core concept in planning projects... It may be included in certain functions aimed at improving service efficiency, yet it will not be specifically highlighted.” | Healing is not explicitly mentioned in planning practices |  |  |  |
| N9: “When people talk about 'healing', national parks are often not their first choice, nor is there adequate public awareness of their healing potential... There is a need to strengthen science popularization to help the public understand the values and experience approaches of national parks. Only in this way can national parks become one of the options for psychological healing in the future; and at the planning and design level, healing can also be truly valued rather than just mentioned in passing.” | The general public does not recognize the healing value of national parks | Public Awareness and Concept Popularization |  |  |
| N10: “In our work, we more frequently use the terms 'ecological well-being for the people', 'public services', and 'sense of gain'. A national park is first and foremost for strict protection, yet protection does not mean isolating people from nature; instead, it means organizing the relationship between humans and nature in a more scientific and sustainable manner.” | Interpreting healing from the perspective of ecological well-being for the people |  |  |  |
| N10: “The ecological protection red line must not be crossed in the name of the so-called 'healing'; public services must not be distorted into over-exploitation and excessive tourism-oriented development; it is imperative to emphasize the principle of inclusiveness, ensuring that such services are not limited to a small number of people or specific locations only.” | Upholding the ecological protection red line and the bottom line of inclusiveness | The Boundaries and Principles of Healing Practices |  |  |
| N2: “I will require the project to have a certain capacity for self-financing. For governments and local residents, if developing the healing industry becomes a burden instead, it would be better to return to the fundamental purpose of national parks, namely biological conservation.” | The healing industry should be beneficial and harmless to local communities |  |  |  |
| N1: “Existing studies often start with the perspective of 'five-sense' experiences... Improvements in water bodies, floral resources, and greening levels can significantly enhance restorative benefits... Currently, the widely recognized positive landscape elements mainly revolve around water features, flowers, layered landscape textures, as well as multi-sensory dimensions such as sound and smell, which generally manifest as improvements in positive emotions and physiological indicators.” | Environmental elements, including water bodies and floral resources enhance healing benefits | Positive Healing Landscape Elements | **Multisensory Stimuli in the Natural Environment** | It is emphasized that natural healing experiences can be strengthened through multi-sensory environmental elements (e.g., visual, auditory, and olfactory stimuli). The mechanisms through which different sensory cues—including pleasing landscapes, natural sounds, and fresh scents—contribute to psychological and physiological recovery are also examined. |
| N1: “Vision remains the primary channel for information acquisition... Over 80% of human information is obtained through vision... Landscape experiences tend to be dominated by visual perception; nevertheless, humans' overall perception of the environment is a synthesis of multiple senses.” | Vision-dominated and multi-sensory integration | Vision-dominated and Multi-sensory Synergy |  |  |
| N2: “Based on my experience, the order of importance is roughly as follows: vision, smell, and hearing. Vision is certainly the primary sensory channel.” | Vision, smell, and hearing are important in that order |  |  |  |
| N2: “I prefer the smell of fresh air, such as the mixed scent of soil and plants. Breathing in such air makes my lungs feel comfortable, and this physical comfort further lifts my mood and fosters a more positive mindset.” | The olfactory soothing effect of fresh air | The Importance of Soundscapes and Olfactory Experiences |  |  |
| N9: “Vision is undoubtedly ranked first. In my opinion, the second place goes to microclimate. Excessively high or low temperatures can both affect one's mood... A relatively ideal scenario is to create a microclimate environment that is warm in winter and cool in summer with comfortable bodily sensations.” | Microclimate comfort is second only to vision |  |  |  |
| N9: “Soundscapes. Many people visit national parks to seek a quiet space for solitude and reflection. Besides anthropogenic noise, the chirping of flocks of animals and the sounds of insects can also be irritating. These sounds can certainly bring a sense of healing on some occasions, but they still require a balance of 'moderate intensity'.” | The balance between quiet natural sounds and noise |  |  |  |
| N13: “Current master plans may still prioritize visual elements, but they have also gradually incorporated certain auditory experiences, such as the sounds of animals or water features... The primary consideration remains focused on visual and soundscapes.” | Soundscapes have begun to be valued in planning |  |  |  |
| N8: “... 'Animals' are actually a very important healing element... The experience of encountering wild animals naturally in their native habitats is entirely different from seeing them in a zoo.” | The encounter with wild animals brings surprise and healing effects | The Healing Experience Brought by Wild Animals |  |  |
| N10: “The first principle is 'zonation and demarcation'. Not all areas are suitable for opening to the public, let alone for visitors to stay. Only when spatial hierarchies such as core conservation, ecological restoration, science popularization and experience, and recreation services are clearly defined can we talk about scientific planning of trails.” | Spatial zonation and classification for differentiating the degree of openness | Zonal Visitor Flow Control and Order Management | **Route Rhythms** | By regulating the rhythm and frequency of spatial use in planning, sustained and balanced interactions between humans and nature can be promoted. This involves delineating functional zones and managing the volume and pacing of visitor flows, enabling people to meet experiential needs while safeguarding ecosystem recovery cycles. |
| N10: “The second principle is 'accessible yet controllable'... to enable the public to access nature without increasing ecological pressure or safety risks, systematic consideration must be given to trail gradients, pavement conditions, detour and exit routes, as well as emergency accessibility.” | Provide controlled accessibility |  |  |  |
| N10: “The third principle is 'controlling major impacts through small-scale measures'... Using such small-scale interventions as trail systems, the placement of stopping points, sightline organization, shading and rain shelter, we can guide visitor flows to carrying-capacity zones and reduce disturbances to sensitive areas.” | Guide visitor flows through small-scale interventions |  |  |  |
| N10: “The general approach is 'intensive management of a small number of key spots + moderately dispersed light-stop points'... Key landscape nodes require intensive management and visitor capacity control... Light-stop points, on the other hand, serve to distribute the flow rhythm, allowing people to slow down and take a break without crowding into a single spot. The combination of the two not only balances visitor experience but also facilitates better management.” | Intensive control of key nodes plus flow dispersion at minor nodes | Stopping Nodes and Flow Rhythm Dispersion |  |  |
| N2: “Natural environments themselves possess a certain capacity for self-restoration... For this very reason, we must better control visitor flows to allow ecosystems to have a recovery period, enabling them to slowly self-regulate and return to a better pristine state.” | Allow intervals for ecological self-restoration | Ecological Recovery Cycles and Visitor Flow Management |  |  |
| N5: “It was tranquil and almost free of litter when the area was not open to the public; even though a host of measures have been implemented after opening, litter still turns up... Children would exclaim in surprise, 'Why is there litter here?' Yet it is truly difficult to achieve full control as visitor numbers grow.” | Increased visitor flows bring environmental disturbances |  |  |  |
| N9: “It is hoped that a pilot program will be launched focusing on 'stopping rhythm': observe the duration of stay and behaviors of people at specific locations, so as to determine which environmental elements or activity types may generate healing effects, and gradually identify the effective elements through elimination and classification methods.” | Identify healing elements based on stay duration | Pilot Exploration and Stopping Behavior |  |  |
| N1: “At the operational level, the first priority is visitor capacity control to avoid noise disturbances... Secondly, we should enhance greening and reduce interferences through visitor flow organization... Use plant barriers to absorb noise; water bodies can provide a background of 'white noise'... Create diverse ecological environments, improve biodiversity, attract birds and insects, and enrich the natural soundscape. Overall, the focus of the strategy still mainly revolves around the two dimensions of vision and hearing.” | Control visitor capacity and mitigate noise via greening to create a multi-sensory environment | Capacity Management and Visitor Flow Diversion | **Management and Operational Strategies** | Natural healing goals should be ensured through management and operational strategies, including visitor capacity control, zonal guidance, visitor flow organization, interpretive education, and staff training, to provide a high-quality healing experience while safeguarding ecological integrity, safety, and order. |
| N10: “Capacity management and time-slot reservations. Crowding can lead to noise, conflicts and boundary violations... Organize visitor flows through zonal and linear division, and appropriately separate express routes and check-in routes from slow-traffic routes and quiet routes... Shift interpretation methods from an 'indoctrination-based' approach to a 'guidance-based' one, which can not only clarify ecological boundaries, but also guide the public to develop low-impact behavioral habits. The ultimate goal is to enable everyone to 'see the rules, understand the rules and be willing to abide by the rules'.” | Visitor flow limitation and reservations, visitor flow diversion, and guidance-based interpretation | Capacity Management and Visitor Flow Diversion / Interpretive Guidance and Normative Education |  |  |
| N5: “For example, a certain national park conducts a wide range of training programs: training is provided to rangers and scenic area staff... Visitors are continuously informed of rules, such as the prohibition of driving vehicles into the park... Signage emphasizes that rare species must not be picked; it also highlights the 'Three No’s Principle': do not disturb, do not feed, and do not approach... Visitors are told that this not only protects animals, but also protects humans themselves... As a result, visitors are more receptive to these rules and more willing to abide by them.” | Train staff and disseminate ecological rules |  |  |  |
| N5: “I believe it is a two-way process... If we train local mentors and engage rangers and other staff in visitor guidance after proper training, it can also bring them an additional source of income. More importantly, the public will develop a better understanding of the value of ranger work and national parks. For urban residents... gaining access to high-quality natural environments... can help them truly relax and escape the pressures of urban life. Overall, it is a win-win situation for both parties.” | Train local rangers to act as guides and achieve a win-win outcome | Staff Training and Local Participation |  |  |
| N6: “Most healing experiences do not start upon entry, but from the moment of arrival, queuing, parking, and entrance services. The orderliness, commercial intensity, and noise control of the entrance community can either raise or lower visitors’ psychological baseline. Another factor is the approach to 'ranger-public communication', which determines whether visitors feel regulated or cared for.” | Regulate entrance order and manage service experience | Entrance Management and Integrated Coordination |  |  |
| N1: “It is necessary. At this stage, research on the restorative benefits of national parks is still relatively preliminary. Research and service strategies can be differentiated according to different age groups; in particular, for the elderly, it is necessary to collect information on their past medical history in a more systematic manner.” | Differentiate strategies for distinct age groups and pay attention to the health background of the elderly | Design Requirements for Specific Groups | **Equity and Needs for Diversity** | Design and service provision should attend to the natural healing needs of diverse groups (e.g., older adults, children, people with disabilities, and those from different cultural backgrounds). Under the premise of ecological protection, inclusiveness and equity should be strengthened to ensure that natural healing benefits all population groups. |
| N10: “The overarching principle is 'safer, more accessible, and more controllable'. For the elderly, it is necessary to address the needs of continuous accessibility, adequate rest stop density, clear risk reminders, and emergency access. For children and families, the main concern is caregiver burden, so it is essential to design more distinct visitor flows and more predictable hazard points. Group tourists tend to generate excessive noise and cause overcrowding, which can be mitigated through time-based and zonal management.” | Differentiated arrangements for the elderly, children and group tourists |  |  |  |
| N2: “From the perspective of social equity, the only feasible approach is to make compensatory efforts in operation and services—for instance, developing a dedicated, relatively safe route for people with limited mobility... to strive to provide them with opportunities to access nature. However, if large-scale construction of barrier-free facilities for the sake of mobility convenience results in severe damage to the area, I would rather abandon further development. Humans have already occupied most of the Earth’s space; this pure land should be left more for flora and fauna.” | Trade-off between barrier-free accessibility and ecological protection | Balance between Barrier-free Access and Ecological Protection |  |  |
| N10: “Services should not be limited to a small number of people or a handful of spots.” | Provide inclusive healing services | Principles of Inclusiveness and Equity |  |  |
| N4: “I believe it also lies in 'cultural connotations'... From an American perspective, national parks place greater emphasis on vast, rugged wilderness and primitive charm; whereas for China’s national parks, the integration of cultural ideas is indispensable. People with different educational backgrounds and regional cultural identities may have distinct mindsets when experiencing national parks.” | Cultural backgrounds shape the mindsets of healing experiences | Cultural Contextual Differences |  |  |
| N1: “Our research approach is to gradually transform the originally qualitative content into a quantifiable and verifiable evidence system... In the early stage of natural healing research, basic methods such as questionnaires were commonly used; in recent years, a growing number of studies have begun to align with cutting-edge international technologies and methodologies... Based on the evidence accumulated so far, blue-green spaces have a certain auxiliary healing effect on reducing public health expenditure and promoting the physical and mental recovery of urban residents.” | Transform qualitative research into quantitative research and verify the therapeutic effects of blue-green spaces | Evidence Accumulation in Natural Healing Research | **Evidence Base and Outcomes Evaluation** | It is emphasized that natural healing practices should be grounded in scientific evidence and that outcomes should be monitored and evaluated. This includes developing quantitative indicators, employing experimental and observational methods, and establishing an integrated monitoring and evaluation system to verify and improve the effectiveness of natural healing. |
| N3: “It can be divided into two levels: the first is 'immediate recovery', and the second is 'health benefits derived from behavioral dosage'. What you need to do is 'identification of design elements', and the key is to align these elements with the mechanisms underlying the two levels.” | Distinguish between two-tier outcomes: immediate recovery and long-term health benefits | Monitoring Indicators for Healing Outcomes |  |  |
| N1: “It depends on the scale of the research objects. If it is a small-scale environment, it is feasible to conduct comparative studies by manipulating variables... If it is a more macro-level strategy evaluation, correlation analysis can be carried out between indicators such as green space ratio and disease rates.” | Small-scale experimental comparison vs. macro-level correlation analysis |  |  |  |
| N1: “In the context of national parks... to evaluate healing experiences, a combination of the following methods can be adopted: first, tourists' subjective scales and feedback on perceptions; second, behavioral data... third, monitoring physiological indicators using wearable devices for specific groups of people.” | Comprehensive evaluation combining subjective questionnaires, behavioral data, and physiological monitoring |  |  |  |
| N9: “I prefer behavioral indicators, such as dwell time and path changes. Physiological indicators often require a longer verification period, are difficult to implement, and are also susceptible to external interference; although subjective psychological scales can reflect perceptions, self-reported data are subjective and their credibility needs to be treated with caution. In comparison, behaviors are more objective... Through the analysis of dwell time and behavioral trajectories, it is easier to judge whether the healing effect is effective.” | Behavioral data are more objective and reliable, while physiological and subjective indicators are subject to limitations | Trade-off between Behavioral and Physiological Indicators |  |  |
| N10: “From a management perspective, I would recommend establishing a 'two-tier indicator system'. The first tier consists of universal governance indicators applicable to all national parks, including passenger flow peaks and troughs, route congestion levels, visitor stay duration, complaints and safety incidents, trespassing frequency, and noise levels... The second tier comprises experience and health-related indicators to be piloted in parks with mature conditions... Indicators such as subjective recovery perception, emotional scales, and wearable-device-derived heart rate variability can be piloted and verified on the premise of small sample sizes... In addition, it is crucial to integrate these indicators into a unified monitoring and evaluation system, forming a closed loop of 'monitoring – analysis – scheduling – feedback'.” | A two-tier evaluation system comprising universal governance indicators and experience-health indicators | Exploration of Indicator System Construction and Standardization |  |  |
| N1: “If we are to conduct healing-related research, I would prefer to start with big data: for example, conducting preliminary macro-level analysis by correlating indicators such as the pilot area and greening level of national parks with data like the disease incidence and hospital admission records of surrounding populations. This can then guide more in-depth, micro-level research subsequently. After all, national parks cover a vast scale, making it relatively difficult to directly carry out point-to-point research.” | Conduct preliminary demonstration with macro-level big data and then guide in-depth micro-level research |  |  |  |
| N12: “Among the natural healing projects we have been involved in, few have been truly implemented... The healing concept is taken into consideration during the design phase, but we have not conducted long-term tracking of the usage effects from the design perspective... Our understanding is that compared with outdated environments, new-type healing spaces offer a more user-friendly experience and may exert certain positive effects on rehabilitation.” | The healing effects of actual projects lack tracking and verification | Limitations of Evaluation Practices |  |  |
| N12: “It is still a planning project that remains at the stage of a long-term vision, without being refined to the point of quantifying the benefits and returns. Therefore, the effectiveness monitoring indicators have not been designed in detail either.” | Healing effectiveness indicators have not yet been specified at the planning stage |  |  |  |
